# Picking up speed: cell cycle regulation during effector CD8^+^ T cell differentiation

**DOI:** 10.1007/s00430-023-00768-7

**Published:** 2023-06-06

**Authors:** Lorenz Kretschmer, Noémie Fuchs, Dirk H. Busch, Veit R. Buchholz

**Affiliations:** 1 Institute for Medical Microbiology, Immunology and Hygiene, Technische Universität München (TUM), Munich, Germany; 2 German Center for Infection Research (DZIF), Partner Site, Munich, Germany

**Keywords:** Immunological memory, CD8^+^ T cells, T cell memory, Clonal expansion, Single cell fate mapping, Cell cycle

## Abstract

Clonal expansion and development of immunological memory are two hallmarks of adaptive immune responses. Resolving the intricate pathways that regulate cell cycle activity and lead to the generation of diverse effector and memory T cell subsets is essential for improving our understanding of protective T cell immunity. A deeper knowledge of cell cycle regulation in T cells also has translational implications for adoptive cell therapies and vaccinations against infectious diseases. Here, we summarize recent evidence for an early diversification of effector and memory CD8^+^ T cell fates and discuss how this process is coupled to discrete changes in division speed. We further review technical advances in lineage tracing and cell cycle analysis and outline how these techniques have shed new light on the population dynamics of CD8^+^ T cell responses, thereby refining our current understanding of the developmental organization of the memory T cell pool.

## Introduction

Protective T cell immunity against intracellular pathogens critically depends on the activation of rare naïve CD8^+^ T cells, their subsequent clonal expansion and differentiation into functionally diverse effector and memory subsets [1, 2]. Naïve CD8^+^ T cells become activated when they first encounter their cognate antigen, presented on major histocompatibility complex-I (MHC-I) molecules on the cell surface of antigen-presenting cells, and integrate further activating signals via co-stimulatory molecules and inflammatory cytokines [2, 3]. Following activation, naïve T cells switch from a relatively quiescent to a highly active cell state, enabling them to complete up to 15–20 cell divisions within 7–8 days after infection [1]. During this process, proliferating T cells also differentiate into short-lived/terminal effector cells (SLECs/TEs) as well as long-lived memory precursors (MPs), encompassing central memory precursors (CMPs) and effector memory precursors (EMPs) [2, 4]. These subsets clearly differ in their epigenetic and transcriptional regulation, their migratory properties and functions [2, 4, 5]. TEs elaborate potent antimicrobial effector functions, are specialized in killing infected cells, but become apoptotic once the pathogen is cleared. In contrast, MPs may lack direct killing properties, but give rise to memory T cells that persist in absence of antigen through exceedingly rare homeostatic cell divisions, estimated to occur only once every 450 days in humans [6]. Upon antigen re-encounter memory T cells respond by rapid secondary proliferation and effector cell differentiation, thereby mediating enhanced immune protection against re-infection. The unique properties of memory T cells have made them prime targets for novel vaccination strategies [7] and adoptive T cell therapies (ACT) [8, 9]. However, our current understanding of the developmental pathways leading to effector and memory T cell formation remains incomplete. Here, we summarize how recent technological advances in in vivo fate mapping and cell cycle analysis provide new insights for better understanding the generation and maintenance of the memory T cell compartment.

## Developmental relationship of effector and memory CD8^+^ T cells

**Fig. 1:**
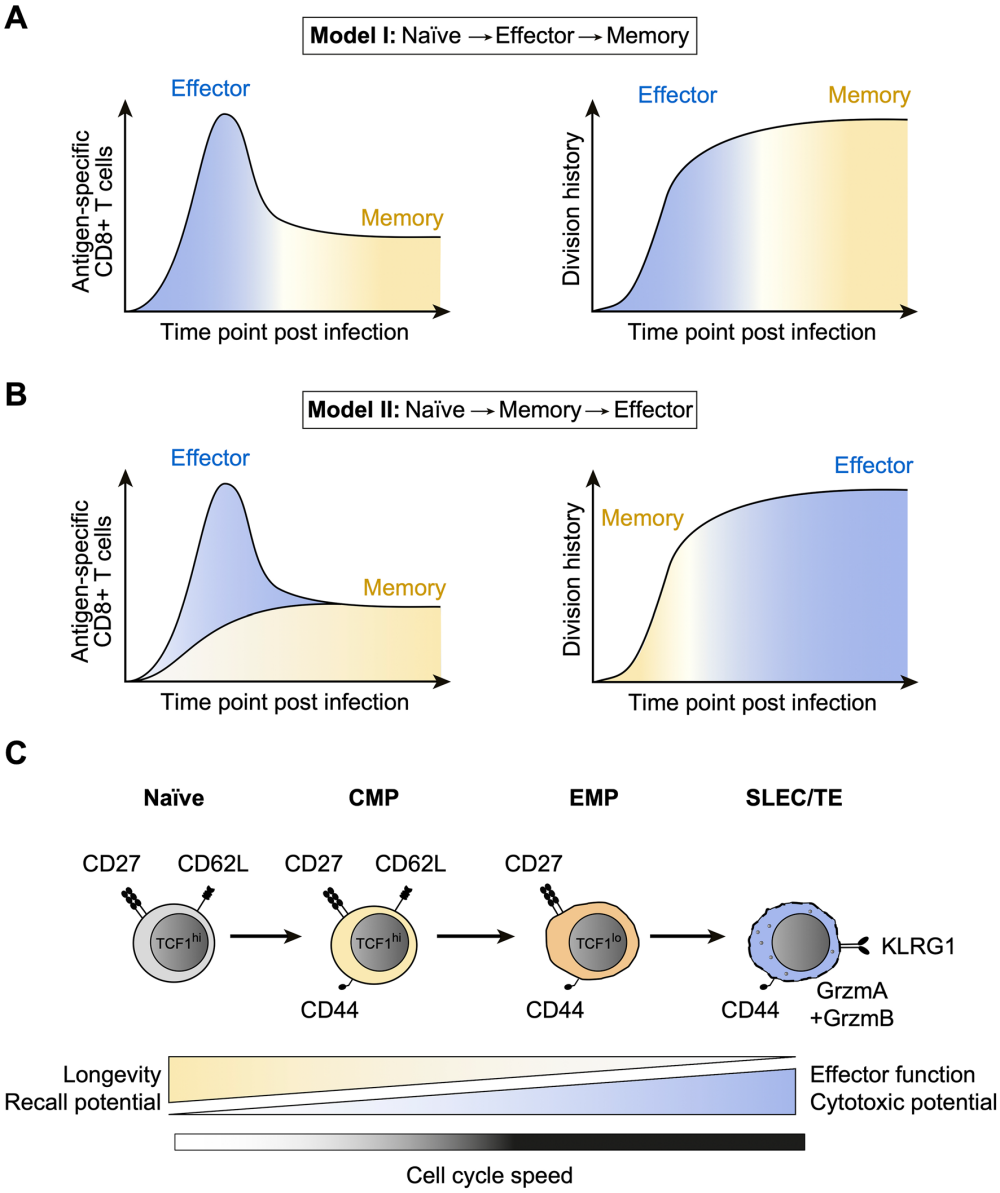
Competing models of memory T cell differentiation. **A** Plots depict the clonal expansion of antigen-specific CD8^+^ T cells in response to infection and their differentiation from naïve to effector to memory cells (Model I, left), as well as the predicted division histories of effector and memory subsets (right). **B** As in **A**, but showing the differentiation of naïve CD8^+^ T cells first into memory and then effector cells (Model II). **C.** Progressive model of CD8^+^ T cell differentiation, based on in vivo single cell fate mapping and population-derived data. Selected markers characterizing naïve, central memory precursor (CMP), effector memory precursor (EMP) and short-lived/terminal effector (SLEC/TE) cells are shown, as are the distinct functional properties of these subsets and a predicted increase of cell cycle speed upon transition from naïve to CMP, EMP and SLEC/TE cells

Despite considerable efforts directed towards characterizing the cellular identities and functional roles of distinct CD8^+^ T cell subsets, controversy remains whether memory cells differentiate either from effector or naïve cells (Fig. 1A–B, left panels) [4, 10]. Depending on either developmental pathway, memory cells are predicted to adopt division histories that largely overlap with or fundamentally differ from those of effector cells (Fig. 1A–B, right panels) [10]. Based on these considerations, resolving the developmental relationships and proliferation activities of distinct T cell subsets not only sheds light on each of these processes separately, but also on how these influence one another, respectively.

Various observations originally favored a differentiation model from naïve to effector and finally memory cells. First, longitudinal analyses of antigen-specific CD8^+^ T cell populations have shown that cytotoxic effector functions are acquired during the early stages of clonal expansion [11, 12], whereas memory cells only begin to predominate and develop recall capacity when the bulk of effector T cells has contracted [13]. Second, fluorescent reporters of effector (*Gzmb*) [14] or SLEC/TE lineage defining genes (*Klrg1*) [15] indicated that a considerable fraction of memory T cells express effector properties at some point during their development. Based on these findings, it has been suggested that memory T cells derive linearly from an expanded pool of effector CD8^+^ T cells and start to emerge around the peak of a primary immune response [16, 17]. In support of this concept, it was recently shown that epigenetic remodeling facilitates the re-expression of naïve or memory-associated genes in antiviral CD8^+^ T cell populations after pathogen clearance [18]. However, it has also been shown that MPs are present throughout most of the primary expansion phase and only constitute a minor subset (e.g. 2–5% CMPs at day 8 after infection) of the overall expanded CD8^+^ T cell population [19–21]. Therefore, it is not clear in how far the epigenetic signatures of such numerically underrepresented subsets were adequately captured by means of bulk profiling. In contrast, novel single cell technologies hold the potential to measure multiple layers of gene regulation in an unbiased manner, while simultaneously capturing the full heterogeneity of antigen-specific T cell populations [22]. This can be used to identify characteristic gene expression signatures of distinct T cell subsets, thereby fostering a more comprehensive understanding of cellular identity and moving beyond simplified classifications based on a small set of established markers. Importantly, single cell profiling can also be applied to score cell cycle activity [23] or infer lineage relationships of developmentally connected subsets by means of trajectory inference [24]. This has for example revealed dynamic precursor–progeny relationships in chronic T cell responses [25–27]. Another informative approach is single cell in vivo fate mapping, where immune responses derived from individual naïve T cells are tracked with unique heritable labels (reviewed in [4]) that are passed on to all daughter cells during clonal expansion and effector/memory T cell differentiation. With this approach, initial studies have established that a single naïve T cell can generate a phenotypically diverse offspring, encompassing CMP, EMP and TE cells, in response to a bacterial infection [28, 29]. Despite such fundamental diversity of single cell-derived T cell responses, the overall magnitude of clonal expansion and the content of CMPs, EMPs and TEs within individual T cell families differed substantially [30, 31]. In particular, an increased clonal output of single T cells can be consistently linked to a relative decrease in CMP phenotype. Computational modeling incorporating the diverse response patterns of individual T cell families suggested two fundamental features of T cell diversification [30]: first, that naïve T cells initially differentiate into CMPs, which then progressively give rise to EMP, and finally TE cells (Fig. 1C). Notably, a similar developmental framework has been proposed for human CD8^+^ T cells [32] and has gained recent support through trajectory inference from parallel bulk mRNA and chromatin accessibility profiling [33]. While these findings conceptually argue for an early fate commitment of activated T cells toward the memory lineage, they do not preclude a transient progression through an effector state at some point during their development. However, some more recent results indeed suggest that central memory (CM) T cells develop without expressing cytotoxic effector functions [34]. The second interesting insight gained from modeling the single cell in vivo fate mapping data is a predicted slower cell cycle speed of CMPs, compared to a more rapid division activity of EMPs and TEs (Fig. 1C) [30], which has important implications for understanding the population dynamics of antigen-specific CD8^+^ T cell responses.

## Diversification of cell cycle speeds occurs early after CD8^+^ T cell activation

**Fig. 2:**
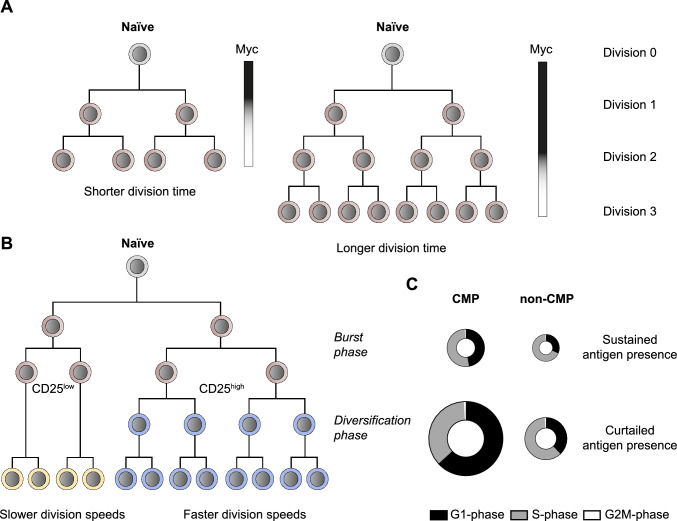
Central memory precursors display slower cell cycle speeds than effector subsets. **A** Schematic representation of CD8^+^ T cell lineage trees with shorter (left) or longer (right) division times, depending on the duration of Myc expression throughout clonal expansion. **B** Lineage tree showing rapid synchronized initial cell divisions (*burst phase*), with emergence of CD25^low^ and CD25^high^ cells, followed by adoption of slower and faster cell cycle speeds (*diversification phase*). Slower dividing cells show a high CMP potential (yellow), whereas faster dividing cells have a high effector cell potential (blue). **C** Plots showing the cell cycle characteristics of CMP and non-CMP cells, measured at day 4 after vaccination in vivo, in presence (upper panel) of absence of sustained antigen availability (lower panel). Colors indicate cell cycle phases and circle sizes indicate overall cell cycle length

The coordinated regulation of cell divisions is central for generating sufficient numbers of antigen-specific CD8^+^ T cells [1, 35] and, immediately after activation, ultra-rapid interdivision times of 2–4 h have been reported [36]. T cells thereby arguably rank among the fastest proliferating cell types in the mammalian organism. However, this rapid division activity has also made it challenging to link the earliest fate decisions of activated T cells to direct changes in their cell cycle. Seminal studies have established that activated T cells undergo up to eight rounds of cell divisions and differentiation into functional effector and memory cells, after few hours of TCR stimulation in vitro [37, 38]. This programmed proliferation activity can be extended in presence of further antigenic stimulation, as well as co-stimulation and cytokines. Elegant work has shown that distinct activating stimuli add linearly to amplify T cell proliferation through a mechanism by which synchronized rapid divisions are followed by an abrupt division cessation [39]. On a molecular level, this is achieved by the timed decay of the cell cycle regulator Myc, which mediates cell cycle exit once expression levels fall below a discrete threshold (Fig. 2A) [40]. These findings highlight the stimulatory requirements through which CD8^+^ T cells initiate and sustain clonal expansion shortly after activation. Additional insights into the subsequent stages of clonal expansion have recently been gained by continuous live cell imaging of single T cells over prolonged in vitro cultures of 4–5 days [41]. This revealed that the initial burst-like proliferation phase, with highly synchronized rapid divisions, is succeeded by a diversification phase, in which slower and faster dividing T cell subsets emerge (Fig. 2B). Interestingly, differential expression of the interleukin-2 (IL-2) receptor alpha chain (CD25) during the burst phase foreshadowed the subsequent diversification of division speeds, with CD25^high^ cells adopting faster cell cycle speeds than CD25^low^ cells. This is notable, since CD25 has been implicated in facilitating effector T cell differentiation [42]. In contrast, more slowly dividing CD25^low^ T cells express CD62L, a key marker of CMP cells (Fig. 2B) [41]. Collectively, these in vitro experiments implicate an intricate link between early cell cycle diversification and effector/memory T cell fate specification. However, the stimulatory conditions during an ongoing infection in vivo are more complex and activating stimuli, such as antigen and inflammation can persist throughout the larger parts of the expansion phase.

## CD8^+^ T cell subsets emerging in response to infection adopt distinct cell cycle speeds

**Fig. 3:**
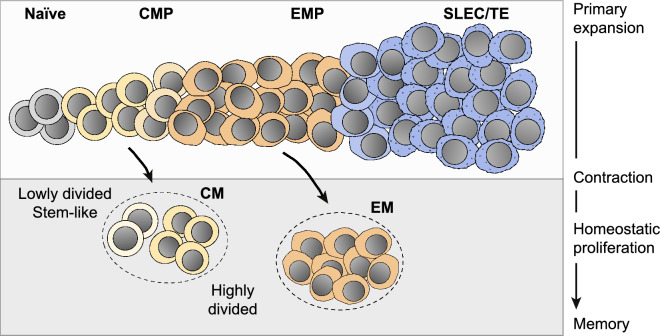
Replication history marks functional heterogeneity in the central memory T cell pool. Schematic representation summarizing the development of antigen-specific CD8^+^ T cell populations at distinct phases of the immune response. The degree of cumulative cell divisions within the central memory (CM) T cell pool marks functional heterogeneity, with lowly divided cells showing stem-like capacity and enhanced responsiveness upon secondary stimulation

To gain further insights into the development of effector and memory T cell subsets, fluorescent reporter systems have been used to measure the cell cycle distribution of antigen-specific CD8^+^ T cells in response to an influenza virus infection [43]. This revealed that a subset of proliferating CD8^+^ T cells can be identified one week after infection, which is characterized by reduced cell cycle activity ex vivo as well as a CMP phenotype. Moreover, novel techniques have recently been developed to measure cell cycle speed directly in vivo and have shed new light on developmental features of adaptive immune responses. One approach is based on administering one or two distinct nucleoside analogs (NAs), that are incorporated into newly synthesized DNA, and measuring total cellular DNA content at defined endpoints [21, 44, 45]. Whereas DNA analysis accurately delineates the current cell cycle position of individual cells, their differential NA profiles are used to resolve their preceding cell cycle stages relative to S-phase. Cells residing in G1 for the total time period between injecting the first NA and analysis (NA^−^DNA^2N^) can thereby be reliably distinguished from cells that progressed through S-phase and divided before entering a new G1-phase (NA^+^DNA^2N^). Moreover, cells with rapid or slow S phases can be distinguished when using two NAs, and only incorporate the first (NA1^+^NA2^−^) or both NAs (NA1^+^NA2^+^), respectively. Initial studies based on these considerations demonstrated that CD4^+^ T cells help accelerates the division speed of germinal center B cells [45] and that a faster cell cycle promotes the somatic hypermutation of B cell immunoglobulin genes [44]. A further technical advancement makes use of the very short bioavailability of NAs after a single injection in vivo, which amounts to ~ 0.5 h [21]. A deliberate delay in DNA content analysis after NA-pulsing can then be used to measure the fraction of dividing cells within a specific time frame (since injecting the NA). Such quantitative measurements can be used to infer average cell cycle durations of dividing T cell populations and refine the categorization of *fast* and *slow* dividing cells, by actually revealing *how fast* and *how slow* their cell divisions occur. For antigen-specific CD8^+^ T cells, the cell cycle durations of CMPs, and non-CMPs were thus measured four days after immunization with activated dendritic cells (DCs), presenting the cognate antigen, together with a systemic *Listeria monocytogenes* (*L.m.*) infection [21]. Remarkably, whereas non-CMPs divided every 6 h, CMPs divided only every 8 h and showed a delayed progression through the G1 phase (Fig. 2C, upper panel). These results highlight that all subsets overall proliferated very rapidly at the early stages during clonal expansion, however they also show that CMPs consistently adopted slower cell cycle speeds than non-CMPs [21]. Surprisingly, these distinct T cell subsets also showed differential sensitivities for changes in their stimulatory environment. CMPs have been shown to experience higher levels of TCR signaling in vivo [46] and premature depletion of antigen-presenting DCs led to a pronounced slow-down of the CMP cell cycle (Fig. 2C, lower panel), mediated by delayed G1 and S-phase progression [21]. The fact that non-CMPs better maintained their rapid division speeds in absence of antigen was instead linked to a higher inflammation-driven expression of CD25, which provides enhanced sensitivity to IL-2, as an antigen-independent growth factor [21]. This intricate regulation of division speeds by activating signals is relevant, since memory T cell responses were diminished when antigen availability during primary expansion was curtailed [21]. In a vaccination context, this argues for optimizing the kinetics of antigen release in order to generate optimal numbers of memory precursors that translate into enhanced memory responses to secondary stimulation. An additional layer of complexity is formed by the degree of homeostatic cell divisions that memory T cells undergo after primary expansion and pathogen clearance (Fig. 3). For measuring the long-term proliferation activity of CD8^+^ T cells, a genetic division recorder (DR) was recently developed that can chronicle replication history over prolonged time frames of several months [47]. This was achieved by coupling short tandem nucleotide repeats (STR) to an out-of-frame Cre recombinase, which can be retrovirally transduced into CD8^+^ T cells from mice expressing a *lox-stop-lox* red fluorescent protein (RFP) gene cassette. Slippage of DNA polymerase at the STRs occurs at a stable low rate in every cell division and can eventually generate in-frame Cre variants that mediate excision of the stop codon, resulting in genetically stable RFP expression in the dividing cell and all of its progeny. Combining the DR with single cell transcriptomics further revealed a surprising degree of heterogeneity in the CM T cell compartment. Remarkably, a subset of lowly-divided CM T cells showed enrichment for stemness-associated transcripts and preferentially fueled the proliferative response to secondary antigen stimulation (Fig. 3) [47]. By delineating the replicative histories of functionally distinct memory T cell subsets, these findings refine our current understanding of the developmental organization of the memory T cell pool.

## Conclusion

Recent technological advances have provided unprecedented insights into the diversification of CD8^+^ T cell responses. This has allowed the scientific community to better understand the complex differentiation and proliferation processes that are essential for generating protective T cell immunity. In particular, it has become clear that the emergence of distinct T cell subsets is coupled to discrete changes in cell cycle speed, with CMPs cycling more slowly than non-CMPs. Interestingly, slower cell cycle speeds have recently been implicated in protecting dividing cells from genotoxic stress [48] and CMPs have indeed been shown to maintain a higher degree of DNA damage resistance, compared to non-CMPs [46]. It can thus be speculated that the slower cell cycles of CMPs act as a crucial safeguard to prevent critical genome damage, growth arrest and apoptosis, while promoting long-term persistence and functionality in a subset of antigen-specific CD8^+^ T cells. In line with this notion, CM T cells were shown to harbor stem-cell like functions [49] and elicit enhanced recall responses upon secondary antigen stimulation [17]. However, new insights indicate that stem-like properties are enriched in only a subset of weakly-divided, more quiescent CM T cells [47]. Targeting the cell cycle by genetic or pharmacological inhibition could therefore emerge as a promising strategy to produce more durable cell products for cellular therapies [50, 51]. In addition, it will be interesting to detail the intricate gene regulatory networks that control T cell identity and function, as many transcription factors simultaneously regulate the cell cycle (*proliferation*) and expression of memory/effector associated genes (*differentiation*) [27, 52–55]. In addition, measuring cell cycle activity during chronic infections and tumors might transform our concept of T cell fate diversification in settings where long-term immunity depends on active cellular turnover of functionally distinct subsets. Finally, improving and developing new tools to study the cell cycle may open up new avenues to understanding human immune responses and other cell types in the mammalian organism.

## Data Availability

No primary data was included in this review article.
