# Correction to: Picking up speed: cell cycle regulation during effector CD8^+^ T cell differentiation

**DOI:** 10.1007/s00430-023-00772-x

**Published:** 2023-06-21

**Authors:** Lorenz Kretschmer, Noémie Fuchs, Dirk H. Busch, Veit R. Buchholz

**Affiliations:** 1 Institute for Medical Microbiology, Immunology and Hygiene, Technische Universität München (TUM), Munich, Germany; 2 German Center for Infection Research (DZIF), Partner Site, Munich, Germany

**Correction to: Medical Microbiology and Immunology** 10.1007/s00430-023-00768-7

There is a small error in presentation of Fig. 1. The correct figure (Fig. 1) is

**Fig. 1:**
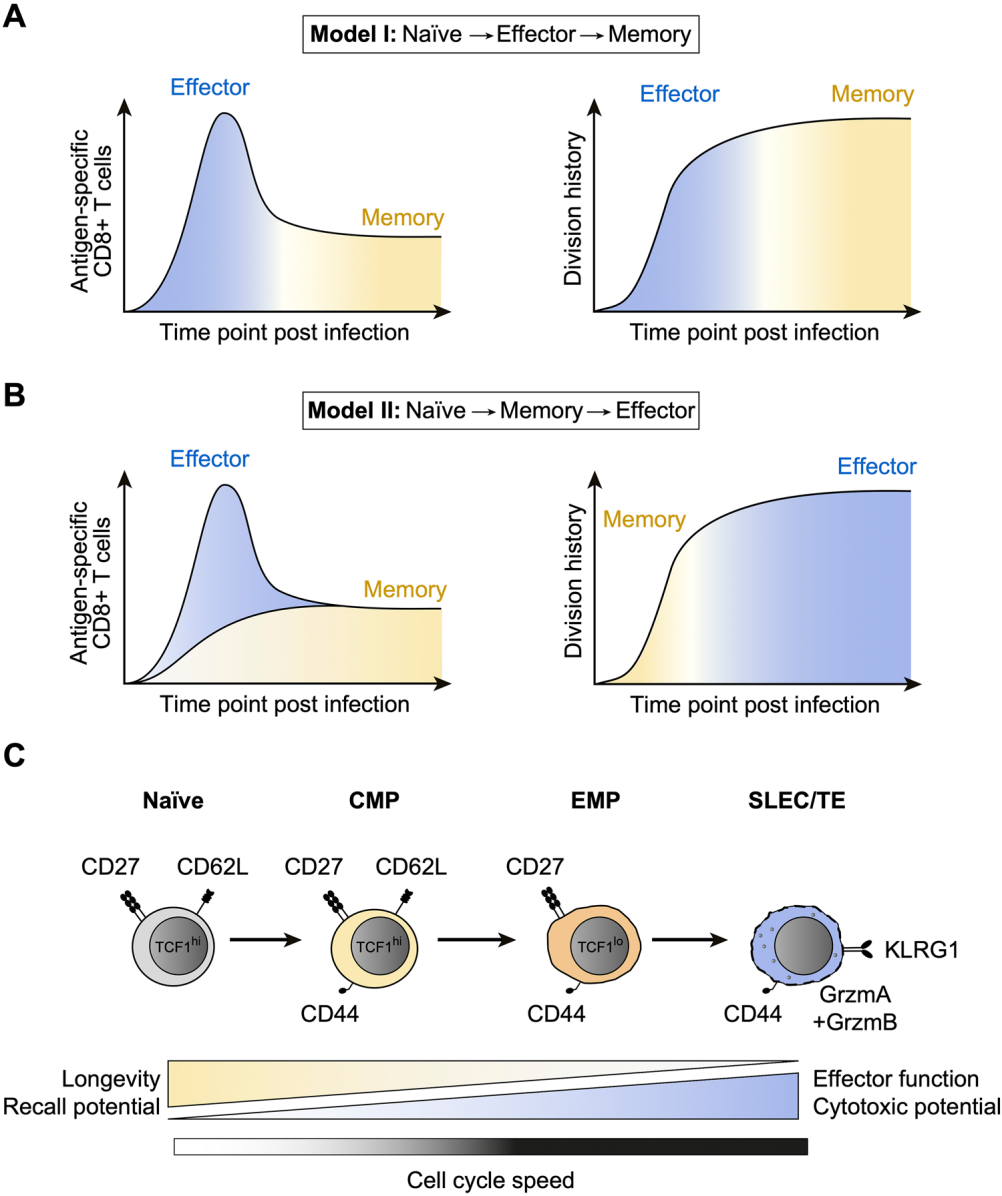
Competing models of memory T cell differentiation. **A** Plots depict the clonal expansion of antigen-specific CD8^+^ T cells in response to infection and their differentiation from naïve to effector to memory cells (Model I, left), as well as the predicted division histories of effector and memory subsets (right). **B** As in **A**, but showing the differentiation of naïve CD8^+^ T cells first into memory and then effector cells (Model II). **C.** Progressive model of CD8^+^ T cell differentiation, based on in vivo single cell fate mapping and population-derived data. Selected markers characterizing naïve, central memory precursor (CMP), effector memory precursor (EMP) and short-lived/terminal effector (SLEC/TE) cells are shown, as are the distinct functional properties of these subsets and a predicted increase of cell cycle speed upon transition from naïve to CMP, EMP and SLEC/TE cells

The original article has been corrected.

